# Unveiling The Myth of High Recurrence Rate of Extracranial Arteriovenous Malformations of The Head and Neck: Systematic Review of Case Reports and Case Series

**DOI:** 10.12688/f1000research.147233.1

**Published:** 2024-06-27

**Authors:** Agustian Winarno Putra, Sagung Rai Indrasari, Camelia Herdini, Danu Yudistira

**Affiliations:** 1 Department of Otorhinolaryngology-Head and Neck Surgery, Faculty of Medicine, Nursing, and Public Health Universitas Gadjah Mada, Sleman, Yogyakarta, 55281, Indonesia; 2 Dr. Sardjito General Hospital, Sleman, Yogyakarta, 55281, Indonesia

**Keywords:** Arteriovenous Malformations, Head and neck, Surgery, Endovascular, Embolization, Recurrence

## Abstract

**Background:**

Arteriovenous malformations (AVMs) in the head and neck pose a challenge in their management due to their local aggressiveness and high recurrence risk. This study aimed to analyze literature on head and neck AVM recurrence post-treatment and identify the most effective strategy with a lower recurrence rate.

**Objectives:**

To analyse existing literature on the recurrence of head and neck AVMs following treatment. Our goal was to identify the most effective treatment option with a lower recurrence rate.

**Methods:**

We conducted a thorough literature search using PubMed, ScienceDirect, and Scopus, from year 2000 to the present. Our analysis focused on key endpoints, specifically the recurrence rates of head and neck AVMs following various treatment approaches.

**Results:**

Out of the initial pool of 108 screened articles, a total of 83 patients were deemed suitable for inclusion in the literature review. The reviewed articles demonstrated that appropriate diagnostic tests were documented in 95% of the included studies. Among the patients, 37.3% had previously undergone interventions and were currently dealing with regrowth masses. Notably, 56.6% of patients underwent a combined approach involving both endovascular and surgical methods, while 25.3% opted for a surgical-only approach, and only 18.1% pursued an endovascular-only approach. The studies showed a promising curing rate of AVMs, with a success rate of 94%, albeit with a complication rate of 32.5%. The average follow-up duration for all patients was 26 months, with a standard deviation of 20.5 months. Out of the 83 patients, 5 experienced recurrence, with single-modality approach. Interestingly, no patients who received a multi-modality of treatments experienced recurrence or regrowth of the AVM mass within the follow up period.

**Conclusion:**

The multi-modality approach outperformed single-modality treatments in preventing AVM recurrence. These findings highlight the importance of a comprehensive and multidisciplinary approach in the management of these complex vascular anomalies.

PROSPERO: CRD42023490871 registered on 17/12/2023

## Introduction

Arteriovenous malformations (AVMs) make up a mere 1.5% of all vascular anomalies, and they are frequently found in the head and neck region (47.4%) as well as the extremities (28.5%). There are two main types of AVMs: focal and diffuse. Focal AVMs appear as soft tissue mass and are generally diagnosed during infancy or childhood. They possess a single arterial feeder, distinct borders, and a nidus. These types of AVMs typically respond well to appropriate treatment. In contrast, diffuse AVMs extend across and tend to disrupt tissue boundaries. They are more often identified in older children and adults. Diffuse lesions are more challenging to treat due to their multiple feeding vessels, necessitating close monitoring and repeated interventions. ^1^^,^^2^

Although AVMs are not malignant, they can be locally aggressive and destructive, leading to complications such as severe disfigurement, ulceration, massive haemorrhage, pain and, in the worst cases, heart failure. These anomalies have tendencies to expand, occasionally undergoing sudden increases in growth, influenced by factors like trauma, hormonal changes, or iatrogenic causes The diagnosis was established based on medical history, physical examination, and usually confirmed by MR-angiography or CT-angiography. ^3^^,^^4^

The treatment of AVMs is still controversial; there are no staging criteria or standardized guidelines, and treatment options vary from a conservative approach to more aggressive strategies. In the past, surgical excision was the predominant approach compared to endovascular embolization therapy for arteriovenous malformations (AVMs). However, the current trend is to limit surgical excision to small, localized AVMs due to the unacceptable risk of significant bleeding associated with the procedure. Endovascular embolization treatment often require multiple sessions for comprehensive closure of the AVM. When endovascular treatment is not followed by a surgical phase, potentially leading to recurrences after the natural degradation of embolic materials. A multidisciplinary approach with a combined treatment based on endovascular embolization and surgical excision is a good compromise and is rated a good choice by several studies. ^5^^–^^7^

In this context, we present the outcomes of various modality treatment for management AVMs in the head and neck. The main objective of this research was to perform a comprehensive analysis of existing literature concerning the recurrence of head and neck AVMs following treatment. Our goal was to identify the most effective treatment option with a lower recurrence rate

## Methods

We conducted an extensive and systematic literature review based on Preferred Reporting Items for Systematic Reviews and Meta-Analyses (PRISMA) guidelines. Our study was registered in PROSPERO: CRD42023490871 on December 17, 2023. We identified suitable studies using the online search engines PubMed, Scopus, and ScienceDirect. The keywords for the search are “Arteriovenous Malformations”, “AVMs”, “Head and Neck”, “Endovascular”, “Surgical”. All clinical studies targeting treatment, outcome, complication and recurrence of AVMs of the head and neck were included in the primary review. Retrospective and prospective English studies, as well as case reports published between January 2000 and October 2023 were included. There were no restrictions as to the country of origin, clinical setting or size of the institution in which the treatments were performed. We did not set a minimum sample size of the studies, as any clinical findings or experience in treatment might be of value to clinicians or future studies. We excluded reports that did not specified the therapy used and follow up period. ^8^ We also excluded patients that were loss to follow up and reported still undergoing treatment. First and second author reviewed the title, abstracts and full-text and discussed which literatures to include and exclude in the review. If authors encountered confusion, a third and fourth author were consulted to establish a consensus.

The data that this study collected including age, gender, location of AVMs, imaging modality, history of previous intervention, treatment modality, follow up period, recurrences and complications. Our analysis focused on key endpoints, specifically the recurrence rates of head and neck arteriovenous malformations following various treatment approaches. As interventional therapy of AVMs of the head and neck details on gender, age, of the treated patients were inconsistently reported, they did not undergo analysis.

## Results

**Figure 1.**
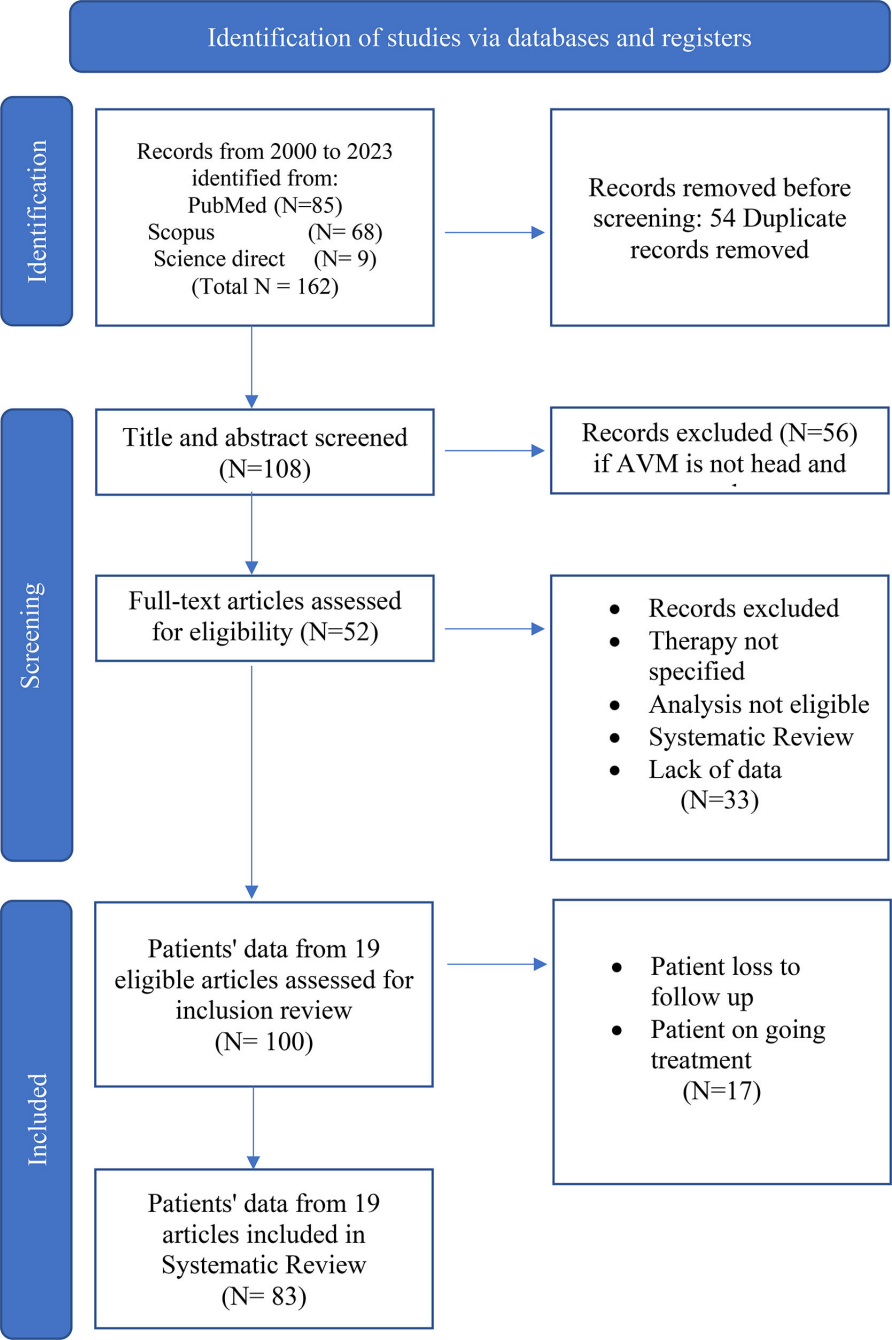
PRISMA flow diagram of the study.

Our search generated a total of 162 citations, and identified a total of 108 potentially relevant articles. Of these articles, a total of 52 articles merited full text review. Based on the above-listed inclusion and exclusion criteria mentioned in our PRISMA figure ( Figure 1), a total of 19 articles were included in the analysis. From the 19 articles, 83 patients ( Table 1) were deemed suitable for inclusion in the literature review.

**Table 1.** Baseline Characteristics.

Study Reference	Age (Years), Sex	Location of AVMs	Imaging Modality	Treatment Modality	Follow up (months)	Recurrance	Complication
Almesberger et al., 2016 ^2^	40, F	Cheek and nose	Doppler ultrasound, angiography, MRI	Embolization and surgery	72	No	Wound dehiscence
	39, F	Nose	Doppler ultrasound, angiography, MRI	Embolization and surgery	72	No	Wound dehiscence
Aslan et al., 2008 ^4^	33, F	Right retro auricula	MRI, CT, angiography	Embolization and surgery	36	No	
Byatnal et al., 2014 ^6^	19, M	Temporal	MRA, MRI, angiography	Surgical	6	No	
Chelilah et al., 2018 ^7^	32, M	Tongue	-	Endovascular	6	No	Oral erosion
	44, M	Right inferior ear and neck	MRI	Endovascular	18	No	
	9, F	Right clavicle	MRI	Endovascular	20	No	Localized erosion, oral erosion
	33, F	Left upper lip and cheek	MRI	Endovascular	8	No	Oral erosion
	16, M	Left cheek, ear and neck	MRI	Endovascular	40	Yes	Pulmonary embolism, oral erosion
	13, M	Forehead	MRI	Embolization and surgery	15	No	Pruritus
Chimona et al., 2005 ^12^	52, F	Left side of the floor of the mouth	MRI, doppler ultrasound, arteriography	Embolization and surgery	24	No	
Cuong et al., 2023 ^13^	46, M	Left ear	Angiography, DSA	Embolization and surgery	7	No	Dry skin
Ferres et al., 2015 ^14^	9, F	Mandibular left first molar	CT, MRA	Embolization and surgery	96	No	
Gennaro et al., 2023 ^5^	48, M	Left cheek	CT, MRI, Angiography	Embolization and surgery	12	No	
	57, F	Left nose wing	CT, MRI, Angiography	Embolization and surgery	12	No	
	27, F	Right cheek	CT, MRI, Angiography	Embolization and surgery	12	No	
	63, M	Inferior lip	CT, MRI, Angiography	Embolization and surgery	12	No	
	28, F	Frontal	CT, MRI, Angiography	Embolization and surgery	12	No	
	39, M	Right cheek	CT, MRI, Angiography	Embolization and surgery	12	No	Cutaneous dyschromia
	37, F	Superior lip	CT, MRI, Angiography	Embolization and surgery	12	No	Lip asymmetry
	40, F	Right cheek	CT, MRI, Angiography	Embolization and surgery	12	No	
	20, M	Inferior lip	CT, MRI, Angiography	Embolization and surgery	12	No	
	24, M	Frontal	CT, MRI, Angiography	Embolization and surgery	12	No	Wound dehiscence
	34, M	Left auricular cervical extending	CT, MRI, Angiography	Embolization and surgery	12	No	Wound dehiscence
	9, M	Superior lip	CT, MRI, Angiography	Embolization and surgery	12	No	
	22, M	Left auricular cervical extending	CT, MRI, Angiography	Embolization and surgery	12	No	
	35, M	Left superior eyelid	CT, MRI, Angiography	Embolization and surgery	12	No	
Han et al., 2015 ^15^	13, M	Lip	Angiography	Embolization and surgery	6	No	
Hosny et al., 2020 ^16^	32, F	Forehead	CTA, doppler ultrasound	Surgical	12	Yes	
	46, M	Below right ear	CTA	Surgical	12	No	
Koshima et al., 2003 ^17^	32, F	Left cheek	Angiography	Embolization and surgery	48	No	
	64, M	Left cervical and temporal region	Angiography	Embolization and surgery	84	No	Temporary facial palsy
Lee et al., 2013 ^18^	25, F	Cheek	MRI, angiography, CT	Surgical	24	Yes	Palsy right side of treatment
Liljie et al., 2022 ^19^	47, M	Nose	MRI, MRA, angiography	Embolization and surgery	72	No	
	12, F	Mandible, floor of the mouth	MRI, MRA, angiography	Embolization and surgery	52	No	
	32, F	Mandible, floor of the mouth	MRI, MRA, angiography	Embolization and surgery	13	No	
	30, F	Mandible, floor of the mouth	MRI, MRA, angiography	Embolization and surgery	38	No	
	18, F	Mandible, floor of the mouth	MRI, MRA, angiography	Embolization and surgery	15	No	
Martines et al., 2009 ^20^	32, F	Base of tongue	CT, Angiography	Endovascular	6	No	Haemoptysis
Pekkola et al., 2013 ^8^	42, F	Tongue	MRI, MRA	Endovascular	11	No	
	20, F	Nose, ala nasion, periorbita	MRI, MRA	Endovascular	24	Yes	
	30,M	Mid and lower face, lower lip	MRI, MRA	Endovascular	30	No	
	12, M	Upper lip	MRI, MRA	Endovascular	11	No	
	15, F	Lower lip	MRI, MRA	Endovascular	5	No	
	17, F	Cheek, submandibular area	MRI, MRA	Endovascular	15	No	
	23, F	Auricle	MRI, MRA	Endovascular	12	No	
	30, M	Midface, upper lip	MRI, MRA	Endovascular	12	No	
	33,F	Auricle, scalp	MRI, MRA	Endovascular	6	No	
Pompa., 2011 ^10^	21, F	Front	MRI, angiography, CT	Embolization and surgery	28	No	
	10, F	Front	MRI, angiography	Embolization and surgery	24	No	
	36, M	Lips	MRI, angiography, CT	Embolization and surgery	24	No	
	41, F	Mandibula	MRI, angiography, CT	Embolization and surgery	6	No	
	15, M	Cheek	MRI, angiography	Embolization and surgery	36	No	
	23, M	Cheek	MRI, angiography	Embolization and surgery	36	No	
	18, M	Lips	Angiography	Embolization and surgery	60	No	
	21, M	Lips	Angiography	Embolization and surgery	60	No	
	8, F	Cheek	MRI, angiography	Embolization and surgery	48	No	
	35, M	Mandible	MRI, angiography, CT	Embolization and surgery	45	No	
	42, M	Neck	MRI, angiography, CT	Embolization and surgery	43	No	
	29, F	Lips	-	Surgical	38	No	
	40, M	Lips	-	Surgical	38	No	
	18, M	lips	MRI, angiography, CT	Surgical	29	No	
	28, M	Nasal dorsum	CT	Surgical	28	No	
	14, F	Lips	-	Surgical	19	No	
	9, M	Front	MRI, angiography, CT	Surgical	15	No	
	6, M	Cheek	MRI, angiography	Surgical	12	No	
	36, M	Front	MRI	Surgical	56	Yes	
Prasad et al., 2004 ^21^	26, F	Right side of nose and forehead	CT, Angiography, doppler ultrasound	Surgical	72	No	
Rajput et al., 2022 ^3^	18, F	Buccinator	MRI, angiography	Embolization and surgery	3	No	wound dehiscence
Richter et al., 2010 ^22^	15	Right tongue, floor of mouth, Retro molar triangle, superior pharynx, tonsil	MRI, angiography	Embolization and surgery	11	No	unable to inflate her cheeks
	11	Base of tongue, floor of mouth, Neck	MRI, angiography	Embolization and surgery	11	No	
	21	Right tongue, face, mandible, floor of mouth, lower lip	MRI, angiography	Embolization and surgery	11	No	
	24	Left tongue, base of tongue, floor of mouth, neck	MRI	Embolization and surgery	11	No	
	8 months	Left tongue tip	MRI, angiography	Surgical	11	No	
	6 months	Right tongue base	MRI, angiography	Surgical	11	No	
	6 months	Base of tongue	MRI, angiography	Surgical	11	No	
	11	Right tongue mid	MRI, angiography	Surgical	11	No	
	13	Right tongue base	MRI, angiography	Surgical	11	No	
	8	Left tongue base	MRI, angiography	Surgical	11	No	
	41	Right tongue base	MRI, angiography	Surgical	11	No	
Ros de San Pedro et al., 2018 ^9^	46, M	Temporal muscle	CT, Angiography	Embolization and surgery	12	No	
	37, M	Temporalq muscle	CT, MRI, Angiography	Surgical	48	No	

CT, Computed Tomography; DSA, Digital Subtraction Angiography; MRI, Magnetic Resonance Imaging.

The reviewed articles demonstrated that appropriate diagnostic tests were documented among the 83 patients, a total of 79 individuals, accounting for 95%, utilized angiography, CT, or MRI, either individually or in combination, for diagnosing AVMs ( Table 2). Among the clinicians, the most preferred modalities were MRI, with 68 cases (82%), and angiography, with 62 cases (75%).

**Table 2.** Percentage of Imaging Modality Used (N=83).

Imaging Modality	Cases (%)
Conventional Angiography	62 (75.0)
Computed Tomography	33 (40.0)
Magnetic Resonance Imaging	68 (82.0)
Digital Subtraction Angiography	3 (4.0)
Doppler ultrasound	5 (6.0)
None reported	4 (5.0)

31 individuals among 83 patients, or 37.3%, had previously undergone treatment. This indicates a recurrence rate of 37.3% among the reported cases of AVMs in the head and neck region. It’s important to note that the treatment methods used were evenly distributed among the endovascular approach, surgical approach, and a combination of endovascular and surgical approaches. with 22.6%, 38.7%, 36.7% subsequently ( Table 3).

**Table 3.** Percentage of patient with previous intervention (N=83).

Treatment Modality	Cases (%)
Endovascular approach	7 (22.6)
Surgical approach	12 (38.7)
Combination Endovascular and Surgical approach	12 (36.7)

Out of the 83 reported patients, the combination of endovascular and surgical approach emerged as the most favored method. We identify notably 47 or 56.6% of patients underwent a combined approach involving both endovascular and surgical methods, while 21 patients (25.3%) opted for a surgical-only approach, and only 12 patients (18.1%) pursued an endovascular-only approach. With an average follow-up period of 26 months, combined approach exhibited a remarkable outcome with a 0% recurrence rate. In contrast, the endovascular approach had a 13.3% recurrence rate, and the surgical approach showed a 14.2% recurrence rate, making the combination approach the most successful in preventing AVM recurrence ( Table 4).

**Table 4.** Percentage of recurrences (N=83).

Treatment Modality	Cases (%)	Recurrences (%)
Endovascular approach	15 (18.1)	2 (13.3)
Surgical approach	21 (25.3)	3 (14.2)
Combination Endovascular and Surgical approach	47 (56.6)	0 (0.0)

Out of the 83 cases, 27 complications were reported, representing a complication rate of 32.5%. The surgical approach alone demonstrated the lowest complication rate, with only 2 cases (9.5%). In contrast, the endovascular approach and the combination of endovascular and surgical approach had complication rates of 40.0% and 40.4%, respectively ( Table 5). The most prevalent complication was wound dehiscence, occurring in 11 out of the 27 cases with complications, making up 40.7% of the reported complication cases ( Table 6).

**Table 5.** Percentage of complications (N=83).

Treatment Modality	Cases (%)	Complication (%)
Endovascular approach	15 (18.1)	6 (40.0)
Surgical approach	21 (25.3)	2 (9.5)
Combination Endovascular and Surgical approach	47 (56.6)	19 (40.4)

**Table 6.** Percentage of complication (N=27).

Complication	Cases (%)
Pruritus	1 (3.7%)
Dry skin	1 (3.7%)
Discoloration	1 (3.7%)
Erosion	5 (18.5%)
Haemoptysis	1 (3.7%)
Wound dehiscence	11 (40.7%)
Asymmetry	1 (3.7%)
Palsy	3 (11.1%)
Pulmonary Embolism	1 (3.7%)
Paresthesia	4 (14.8%)

## Discussion

A clinical diagnosis of arteriovenous malformation is diagnosed through the patient’s medical history, physical examination and supportive examination are crucial especially MRI and angiography. MRI currently serves as the definitive method for assessing the extent of involvement within tissue planes and illustrating flow characteristics. Angiography plays a vital role in revealing the feeding vessels, offering insights into anastomoses with other extracranial or intracranial vessels, and providing details about the venous drainage pattern for ‘super selective’ embolization. During embolization, the focus should be on the nidus or epicenter of the arteriovenous malformation rather than the proximal feeding vessels. ^4^

Complete removal of AVMs is imperative to prevent recurrence. To achieve this, a customized approach must be devised for each patient, taking into consideration the specific boundaries of the lesion. The excision process may involve the removal of three different structures: fascia, muscle, and bone. In cases where preoperative embolization has not been performed, extended resection into apparently normal tissue is advised to ensure the thorough elimination of the AVMs. ^9^

The management of AVMs remains the most debated aspect in the medical literature, with no universally accepted approach. Previously, the treatment methods were evenly distributed among the endovascular approach, surgical approach, and a combination of endovascular and surgical approaches. ^10^ Currently, the preferred treatment involves selectively embolizing vessels combined with surgical resection and subsequent reconstruction of soft tissues. The primary goal of preoperative embolization is to minimize blood loss and enhance the surgical procedure, emphasizing that it should not be seen as a method for reducing the extent of resection. It is crucial not to postpone surgical resection beyond 48 hours after embolization, as the inflammation that ensues makes the hemodynamic benefits ineffective and renders the surgery more challenging. ^10^

Recurrence of AVMs has been reported in as much as 80% of cases following embolization or resection. In cases where the nidus is incompletely removed or embolized, there is a heightened risk of aggressive growth in the remaining lesion, leading to a potential progression risk as high as 50% within the initial five years. ^1^ In recent literature findings, the recurrence rate of AVMs that managed with combined approach of both endoscopic and surgical approach give a remarkable outcome with a 0% recurrence rate. This result most likely because the surgeons are able to obtain a clear surgical vision field and completely excise the AVMs mass margin, as a result of the pre-embolization of the AVMs mass. The presence of a clear margin and minimal bleeding likely contributes to this successful outcome.

Currently, in our literature search, the management of AVMs mass shows a better recurrence rate than previously believed. However the average follow-up period was 26 months. In some literature recurrences have been observed a decade after treatment, highlighting the essential need for extended post-treatment follow-up to ensure timely detection. It’s crucial to acknowledge that the interpretation of the term “cure” varies in the literature, and reported instances of “cure” may be influenced by limited follow-up periods. Some cases defined “cure” as an asymptomatic state following embolization rather than a complete absence of the condition. ^1^^,^^11^

### Study strength and limitations

To our understanding, several studies have documented cases and case series involving patients with AVMs in the head and neck region, who were treated using diverse approaches such as endovascular, surgical, or a combination of both. Nevertheless, as of now, no systematic review has been conducted to determine the most effective treatment approach, particularly in relation to the recurrence of the mass.

The limitations of this study is the lack of randomization due to the rarity of the case we included all studies that met the inclusion criteria regardless of the size of the lesion, race, gender, age and location. Subsequently, even though the average follow-up period was 26 months, the follow up period of the studies varies greatly ranging from 6 month to 8 years. The lack of literature addressing the recommended follow-up timeframe for AVMs mass contributes to the uncertainty. Some studies even report occurrences of AVMs mass recurrence a decade post-treatment. ^11^

## Conclusion

The multi-modality of endovascular and surgical approaches has a lower recurrence rate of AVM masses compared to a single-modality treatment. These findings highlight the importance of a comprehensive and multidisciplinary approach in contributing to the successful surgical outcomes of these complex vascular anomalies.

## Ethics and consent

Ethical approval and written informed consent were not required.

## Data Availability

All data underlying the results are available as part of the article (included under extended data) and no additional source data are required. **Supplementary data**: Figshare: Tabel 1. Baseline Characteristic, https://doi.org/10.6084/m9.figshare.25931002.v2. ^23^ Figshare: PRISMA Checklist https://doi.org/10.6084/m9.figshare.24995798.v2. ^24^ Figshare: PRISMA Flowchart https://doi.org/10.6084/m9.figshare.25783014.v1. ^25^ Data are available under the terms of the Creative Commons Zero “No rights reserved” data waiver (CC0 1.0 Public domain dedication).
