# Supplementary material for: Unveiling The Myth of High Recurrence Rate of Extracranial Arteriovenous Malformations of The Head and Neck: Systematic Review of Case Reports and Case Series
Source: F1000Res. 2024 Dec 24;13:697. Originally published 2024 Jun 27. [Version 2] doi: 10.12688/f1000research.147233.2 (PMC11809641; doi:10.12688/f1000research.147233.2)
Supplement: Peer review history

# Reviewer report: Reviewer response for version 2

**DOI:** 10.5256/f1000research.175581.r352426

**Published:** 2025-01-02

**Authors:** Mustafa Ismail

**Affiliations:** 1 University of Baghdad, Baghdad, Iraq

Thank you for making the required revision. I have no further comments.

Are the rationale for, and objectives of, the Systematic Review clearly stated?

Yes

Is the statistical analysis and its interpretation appropriate?

Partly

If this is a Living Systematic Review, is the ‘living’ method appropriate and is the search schedule clearly defined and justified? (‘Living Systematic Review’ or a variation of this term should be included in the title.)

Yes

Are sufficient details of the methods and analysis provided to allow replication by others?

Yes

Are the conclusions drawn adequately supported by the results presented in the review?

Yes

Reviewer Expertise:

Neurosurgery

I confirm that I have read this submission and believe that I have an appropriate level of expertise to confirm that it is of an acceptable scientific standard.

# Response

**Published:** 2025-01-03

**Authors:** Agustian Winarno Putra

**Affiliations:** Otorhinolaryngology, Universitas Gadjah Mada, Yogyakarta, Special Region of Yogyakarta, Indonesia

Thank you for your review

# Reviewer report: Reviewer response for version 2

**DOI:** 10.5256/f1000research.175581.r352427

**Published:** 2024-12-31

**Authors:** Remco de Bree

**Affiliations:** 1 Utrecht University, Utrecht, The Netherlands

My questions are sufficiently answered. I recommend approval of this manuscript.

Are the rationale for, and objectives of, the Systematic Review clearly stated?

Yes

Is the statistical analysis and its interpretation appropriate?

Yes

If this is a Living Systematic Review, is the ‘living’ method appropriate and is the search schedule clearly defined and justified? (‘Living Systematic Review’ or a variation of this term should be included in the title.)

Yes

Are sufficient details of the methods and analysis provided to allow replication by others?

Yes

Are the conclusions drawn adequately supported by the results presented in the review?

Yes

Reviewer Expertise:

Head and neck cancer

I confirm that I have read this submission and believe that I have an appropriate level of expertise to confirm that it is of an acceptable scientific standard.

# Reviewer report: Reviewer response for version 1

**DOI:** 10.5256/f1000research.161404.r339828

**Published:** 2024-11-26

**Authors:** Mustafa Ismail

**Affiliations:** 1 University of Baghdad, Baghdad, Iraq

The systematic review on extracranial arteriovenous malformations (AVMs) provides valuable insights into recurrence rates following various treatment modalities, with an emphasis on the benefits of a multi-modality approach. However, it could be enhanced by addressing a few points. Firstly, the discussion on the role of reconstruction with well-vascularized tissue after surgical excision should be expanded to explore its potential in minimizing local hypoxia and reducing recurrence. Secondly, the interpretation of cure and recurrence rates might benefit from clarifying follow-up duration variations and their impact on outcomes. Lastly, incorporating long-term follow-up data and analyzing outcomes across diverse demographic groups could further substantiate the findings.

 - Please recheck the PRISMA diagram for No of studies exclusion.

Are the rationale for, and objectives of, the Systematic Review clearly stated?

Yes

Is the statistical analysis and its interpretation appropriate?

Partly

If this is a Living Systematic Review, is the ‘living’ method appropriate and is the search schedule clearly defined and justified? (‘Living Systematic Review’ or a variation of this term should be included in the title.)

Yes

Are sufficient details of the methods and analysis provided to allow replication by others?

Yes

Are the conclusions drawn adequately supported by the results presented in the review?

Yes

Reviewer Expertise:

Neurosurgery

I confirm that I have read this submission and believe that I have an appropriate level of expertise to confirm that it is of an acceptable scientific standard, however I have significant reservations, as outlined above.

# Response

**Published:** 2024-12-15

**Authors:** Agustian Winarno Putra

**Affiliations:** Otorhinolaryngology, Universitas Gadjah Mada, Yogyakarta, Special Region of Yogyakarta, Indonesia

First of all, we would like to thank Mustafa Ismail for  the  detailed review, which has greatly improved this publication

 - Firstly, the discussion on the role of reconstruction with well-vascularized tissue after surgical excision should be expanded to explore its potential in minimizing local hypoxia and reducing recurrence. Secondly, the interpretation of cure and recurrence rates might benefit from clarifying follow-up duration variations and their impact on outcomes. Lastly, incorporating long-term follow-up data and analyzing outcomes across diverse demographic groups could further substantiate the findings.

**We added a publication by Balakrishnan et al (2023.) in the publication it indicates that free flaps, offering a robust and uniform blood supply, are significantly more effective in preventing recurrence compared to pedicled flaps or skin grafts. One study highlighted a 0% recurrence rate with free flaps, in contrast to a 64% recurrence rate with pedicled flaps and grafts. Additionally, patients undergoing reconstruction with free flaps after wide local excision achieved markedly better functional and aesthetic outcomes, with 87.5% reporting good-to-excellent results and no recurrences observed during the follow-up period**

**this evidence is back up with the theory from Lu et al (2011) that stated recurrence of AVMs is often associated with neovascularization driven by factors such as hypoxia-inducible factor-1α (HIF-1α) and vascular endothelial growth factor (VEGF. These factors are upregulated in hypoxic conditions, promoting the recruitment of endothelial progenitor cells and new vessel formatio**n

**While this topic has piqued our interest, we believe it warrants a more detailed review and in-depth discussion in a separate publication.**

 - Please recheck the PRISMA diagram for No of studies exclusion.

**During the revision process, we re-evaluated all the data and found an error in our initial assessment. Specifically, we mistakenly stated that 33 studies were excluded, resulting in 19 studies being included in the final analysis. In fact, 32 studies were excluded, and 20 studies were included in the final analysis. There are no changes to the actual number of final patients eligible for analysis.**

**We apologize for the error in which we incorrectly stated that the patient reported by Ferres et al. (2015) underwent both endovascular and surgical approaches. In fact, the treatment was solely via the endovascular approach using a sclerosing agent.  Additionally, we rearranged the order of patients in the table to match the original publication, making it easier to cross-check the data.**

# Reviewer report: Reviewer response for version 1

**DOI:** 10.5256/f1000research.161404.r326069

**Published:** 2024-09-30

**Authors:** Remco de Bree

**Affiliations:** 1 Utrecht University, Utrecht, The Netherlands

This manuscript is about the recurrence rate after treatment of extracranial AVMs in the head and neck area. This systematic review is well performed and the manuscript is well written.

 The outcomes are focuses on recurrence. Limitations of the study are described.

 I have only three minor points - 

 - 'Currently, in our literature search, the management of AVMs mass shows a better recurrence rate than previously believed'. Please change 'better' in 'lower'.

 - The aim is to emboloize the nidus, which is very difficult. If embolization is the only treatment this is very important. If it is performed pre-operatively it is less important, than embolization of the feeding arteries may be enough.

 - Can the authors discuss the reconstruction of the defect after surgical excision. There are suggestions that reconstruction with well vascularized tissue (thus avoiding local hypoxia) may reduce the risk of recurrence.

Are the rationale for, and objectives of, the Systematic Review clearly stated?

Yes

Is the statistical analysis and its interpretation appropriate?

Yes

If this is a Living Systematic Review, is the ‘living’ method appropriate and is the search schedule clearly defined and justified? (‘Living Systematic Review’ or a variation of this term should be included in the title.)

Yes

Are sufficient details of the methods and analysis provided to allow replication by others?

Yes

Are the conclusions drawn adequately supported by the results presented in the review?

Yes

Reviewer Expertise:

Head and neck cancer

I confirm that I have read this submission and believe that I have an appropriate level of expertise to confirm that it is of an acceptable scientific standard.

# Response

**Published:** 2024-12-15

**Authors:** Agustian Winarno Putra

**Affiliations:** Otorhinolaryngology, Universitas Gadjah Mada, Yogyakarta, Special Region of Yogyakarta, Indonesia

First of all, we would like to thank Remco de Bree the  detailed review, which has greatly improved this publication

 - 'Currently, in our literature search, the management of AVMs mass shows a better recurrence rate than previously believed'. Please change 'better' in 'lower'.

**We already make adjustment a suggested**

 - The aim is to emboloize the nidus, which is very difficult. If embolization is the only treatment this is very important. If it is performed pre-operatively it is less important, than embolization of the feeding arteries may be enough.

 - Can the authors discuss the reconstruction of the defect after surgical excision. There are suggestions that reconstruction with well vascularized tissue (thus avoiding local hypoxia) may reduce the risk of recurrence.

**We added a publication by Balakrishnan et al (2023.) in the publication it indicates that free flaps, offering a robust and uniform blood supply, are significantly more effective in preventing recurrence compared to pedicled flaps or skin grafts. One study highlighted a 0% recurrence rate with free flaps, in contrast to a 64% recurrence rate with pedicled flaps and grafts. Additionally, patients undergoing reconstruction with free flaps after wide local excision achieved markedly better functional and aesthetic outcomes, with 87.5% reporting good-to-excellent results and no recurrences observed during the follow-up period**

**This evidence is back up with the theory from Lu et al (2011) that stated recurrence of AVMs is often associated with neovascularization driven by factors such as hypoxia-inducible factor-1α (HIF-1α) and vascular endothelial growth factor (VEGF. These factors are upregulated in hypoxic conditions, promoting the recruitment of endothelial progenitor cells and new vessel formatio**n

 Thank you
